# Co-Selection of Resistance to Antibiotics, Biocides and Heavy Metals, and Its Relevance to Foodborne Pathogens

**DOI:** 10.3390/antibiotics4040567

**Published:** 2015-11-13

**Authors:** Andrew D. Wales, Robert H. Davies

**Affiliations:** Department of Bacteriology and Food Safety, Animal and Plant Health Agency (Weybridge), Woodham Lane, New Haw, Addlestone, Surrey KT15 3NB, UK; E-Mail: rob.davies@apha.gsi.gov.uk

**Keywords:** biocide, heavy metal, antimicrobial resistance, zoonoses

## Abstract

Concerns have been raised in recent years regarding co-selection for antibiotic resistance among bacteria exposed to biocides used as disinfectants, antiseptics and preservatives, and to heavy metals (particularly copper and zinc) used as growth promoters and therapeutic agents for some livestock species. There is indeed experimental and observational evidence that exposure to these non-antibiotic antimicrobial agents can induce or select for bacterial adaptations that result in decreased susceptibility to one or more antibiotics. This may occur via cellular mechanisms that are protective across multiple classes of antimicrobial agents or by selection of genetic determinants for resistance to non-antibiotic agents that are linked to genes for antibiotic resistance. There may also be relevant effects of these antimicrobial agents on bacterial community structure and via non-specific mechanisms such as mobilization of genetic elements or mutagenesis. Notably, some co-selective adaptations have adverse effects on fitness in the absence of a continued selective pressure. The present review examines the evidence for the significance of these phenomena, particularly in respect of bacterial zoonotic agents that commonly occur in livestock and that may be transmitted, directly or via the food chain, to human populations.

## 1. Introduction

Biocides, in the broadest sense, are substances formulated to be harmful to (or to otherwise control) living organisms [1]. Many authorities adopt more restrictive definitions, similar to those for a microbicide, for the purposes of considering non-antibiotic antimicrobial agents, albeit still with diverse chemical characteristics and applications. Useful definitions in this mould have been provided by Tumah [2] and Sheldon [3], which the authors of the present review regard as reasonable in scope and practicality. With few exceptions (such as iodide salts that show some efficacy in the treatment of certain veterinary fungal and bacterial infections [4]) biocides are not sufficiently selective to be used within body tissues, but some may be used as antiseptics on body surfaces. Other common applications include use as disinfectants on equipment and surfaces in many environments including farms and hospitals, as decontaminants on carcass surfaces following slaughter, as sporicidal sterilants for medical equipment and as preservatives in pharmaceuticals, cosmetics and food [1]. Heavy metals are arbitrarily defined, but commonly-included elements show varying degrees of toxicity for organisms [5,6] with some, such as silver, copper and zinc, being relatively non-toxic to mammalian tissues and systems. These are used as antimicrobial coatings and wound dressings [7,8,9] or (in the case of copper and zinc) in agriculture as in-feed growth promoters and for enteric disease control, particularly in the pig and poultry sectors [10].

Biocidal substances have been used, in one form or another, for centuries [7,11] and the more recent development of versatile biocides with limited toxicity for animal tissues, such as quaternary ammonium compounds (QAC), biguanides and bisphenols, has led to increased use of such compounds to assist improved hygiene [12]. Hygienic practices have been a cornerstone of efforts to control antibiotic use (and associated resistance) in clinical and farm environments. In addition, general hygiene and the in-feed use of copper and zinc have been increased in pig and poultry production to improve disease prevention in the wake of the phasing out of antibiotic growth promoters in some parts of the World, notably Europe [13]. Organic acids and phytochemicals such as essential oils are antimicrobial agents that also have been trialled as in-feed alternatives to antibiotic growth promoters and for pathogen control [14,15,16,17].

In recent decades observations were made among important pathogenic bacteria genera of reduced susceptibility of isolates to commonly-used biocides, particularly in healthcare settings [18,19,20]. This was often seen in association with an elevated frequency of antimicrobial resistances [21,22]. Concerns have consequently been raised that in certain circumstances bacteria may become unsusceptible to some biocide regimes, or that a reduced susceptibility to biocides may foster an elevated degree or frequency of antimicrobial resistance [1,23,24]. Whilst such concerns were initially focussed upon healthcare environments, where biocides and antibiotics were frequently used and where monitoring was commonly practised, the scope of investigations has broadened to include other areas, such as domestic cleaning and hygiene products where the use of biocides has increased [25,26].

A major area under particular scrutiny is the livestock food chain, from farms and hatcheries through slaughter facilities and dairy processing plants to food processing, packaging and retail facilities [1]. This has partly been driven by alarm over evidence of multi-resistant pathogens, including *Salmonella* (pathovar Typhimurium DT104 and latterly monophasic Typhimurium variants) [27,28,29], livestock-associated meticillin-resistant *Staphylococcus aureus* (LA-MRSA) [30] and extended-spectrum β-lactamase *E. coli* [31], on farms and in the food chain. In this context the possible effects of heavy metals have attracted particular attention, owing to the frequent heavy supplementation of copper and zinc in feeds for some animal species.

The present review examines the evidence for reduced biocide and heavy metal susceptibility among pathogens and bacterial communities, for co-selection with antimicrobials and for mechanisms underlying the observed patterns. This is a broad remit, and coverage is necessarily selective, with an ultimate focus on issues in animal production, zoonotic bacteria and the food chain, plus discussion of the notable differences seen between field and laboratory investigations.

## 2. Agents with the Potential for Co-Selection of Antibiotic Resistance

### 2.1. Antibiotics

Antibiotics, and related drugs such as sulphonamides, exhibit high antimicrobial potency and selective toxicity that is sufficient in many cases to allow their use as anti-infective agents within body tissues. This is usually because of a highly specific action on a microbial target [32], which is commonly inhibitory to growth rather than lethal at normal therapeutic concentrations, and which acts in concert with the host’s immune system to resolve microbial colonisation or infection over an extended period [26,33].

Reduced susceptibility of an organism to an antibiotic may be innate, due for example to features of the microbe’s cell envelope, energy metabolism or the presence of an alternative metabolic pathway. Alternatively, reduced susceptibility may be acquired via single- or multi-step mutation that affects the target site and/or the effective concentration of the antibiotic within the cell, or by the acquisition of genetic material encoding a feature such as an inactivating enzyme or an alternative to the target molecule [34].

Bacterial resistance may be promoted by, among other things, sub-therapeutic antibiotic concentrations in certain tissues or organs, such as the gut, and the tendency for antibiotics to induce non-specific mutagenesis in bacteria [13,35]. A key parameter of an antibiotic-microbe combination is the minimum inhibitory concentration (MIC), indicating a concentration of antibiotic preventing growth of the microbe and likely to prove therapeutically effective if achieved in the target tissue(s).

### 2.2. Biocides

By contrast with antibiotics, biocides have more varied applications, do not operate with the benefit of a concurrent immune response, and commonly have to contend with microbes in protected or resistant states, such as in biofilms or organic matter, in nutrient- or moisture-limited environments, or following sporulation. Consequently they typically are intended to be lethal, usually after a single application, and generally have multiple structural and biochemical targets achieving a gross disruptive effect on micro-organisms [12,36,37]. In consequence of the foregoing considerations, “in use” concentrations of biocides typically are multiples of the laboratory-determined minimum lethal concentration and are intended to be rapidly lethal to the target organisms [26,38].

Several recent reviews provide comprehensive comparisons of biocide *vs.* antibiotic actions and susceptibilities [2,12,25,26,34,39,40]. Authors vary, sometimes markedly, in their assessment of the potential significance of altered biocide susceptibility among bacteria. Gilbert and McBain [26] provide a useful glossary of the often confusing, and sometimes misused, terminology in this field. Classification and activity of biocides are well reviewed by McDonnell and Russell [37], whilst Maillard [36] and Ortega Morente *et al.* [12] provide further discussion of known and postulated targets and modes of action for commonly-used biocides.

Mechanisms of biocide action are incompletely understood and appear to be varied, reflecting the diversity of biocides, applications and associated degrees of toxicity for higher organisms. Only one commonly-used agent, the bisphenol triclosan, has a well-documented specific enzyme target: the enoyl acyl carrier protein reductase (FabI and homologues), involved in fatty acid synthesis [41]. Exposure to triclosan and the development of reduced susceptibility is associated with mutation plus upregulation of *fabI* expression [42]. Some workers believe that all observed effects of triclosan at in-use concentrations are mediated through this specific pathway [12], although in *S. aureus* a lack of correlation between growth phase and kill rate of triclosan [43] suggests that less-specific cell damage may also occur, at least in some Gram-positive organisms.

Most biocides when applied at in-use concentrations appear to affect multiple targets, with membrane effects being a common theme. These may manifest as physical disruption and leakage of cell contents, disruption of membrane ion gradients, and/or permeabilising effects resulting in enhanced uptake of the biocide itself into the cell interior [12,26,37,44]. Effects on cytoplasmic components appear to be similarly diverse and to affect multiple targets. Given the centrality of cell membrane effects and the need for biocides to traverse the cell envelope and cytoplasmic membrane, it is perhaps unsurprising that Gram-negative bacteria are generally less susceptible to many biocides than are Gram-positive organisms [2,12], and that some common biocides have lipophilic domains in their molecules, allowing close association with cell membrane phospholipids. These include the biguanide chlorhexidine and cationic surfactants, exemplified by the versatile family of quaternary ammonium compounds (QAC) [37,39].

### 2.3. Heavy Metals

The use, particularly in pig and poultry production, of heavy metal-containing compounds for growth promotion and therapy of intestinal disease potentially selects for micro-organisms with reduced susceptibilities to heavy metals and co-selects for reduced susceptibility to other antimicrobials [10]. Unlike biocides (but in common with antibiotic growth promoters) these compounds are used at inhibitory rather than lethal concentrations, thus potentially allowing resistance to emerge within the alimentary tract and the immediate farm environment at a higher frequency than following biocide application.

However, unlike antibiotic growth promoters, such metals are very persistent in the environment. Heavy metals may accumulate in soil, water and sediments from agricultural practices as well as other sources such as aquacultural and marine antifouling treatments or industrial effluent. In one study in the USA around 90% of in-feed copper and zinc was found to be shed in faeces [45], and it has been estimated in the UK that livestock may in some localities be the major source of environmental contamination by zinc and copper [46]. Thus, such destinations provide further environments (in addition to intestinal contents and faeces) in which co-selection for reduced susceptibilities may occur [6,10,47]. Furthermore, it has been hypothesized that genes coding for reduced susceptibilities to copper and zinc among diverse bacteria have long been selected for in the environment by eukaryote phagocytes such as amoebae, which may use copper- and zinc-mediated bacterial killing mechanisms [48].

The modes of action of copper and zinc when used in pig and poultry feed as growth promoters are not known with certainty and probably involve suppression of bacterial fermentation, but possibly also modulation of host responses to bacterial lipopolysaccharide [49]. Effects on host growth are not consistent and may be dependent on animal age [50,51], whilst permitted inclusion rates vary between regulatory regimes and according to age. In the EU, higher inclusion rates are permitted for young piglets and in short-term “medicinal remedies”, which for inorganic zinc compounds may result in inclusion rates in excess of thirty times basal requirements [10,52]. Generally-permitted levels in the EU are around 3 to 4 times the basal requirements, and at these levels no therapeutic effects on enteric disease would be expected. Supplementation of ruminant feed with metals is much less intensive than for pigs and poultry, and in particular sheep are highly susceptible to copper toxicity [53]. Other heavy metals added to animal feed that may result in elevated environmental deposits include iron, cobalt and manganese [6]. Arsenic is a metalloid element that is still permitted as a therapeutic and growth promoting agent in some territories, although its relationship to altered bacterial susceptibilities to other antimicrobials has been little studied [54].

## 3. Mechanisms Linking Altered Susceptibilities of Bacteria to Antibiotics, Biocides and Heavy Metals

### 3.1. Terminology and Testing

Terms such as “resistance” and “tolerance” have acquired specific technical meanings in the field of antimicrobials, and looser usage is generally discouraged. Current terminology distinguishes between clinical and microbiological antibiotic resistance [55]. Clinical resistance is present when phenotypic testing of a microbe/antibiotic combination against a clinical breakpoint indicates that therapeutic failure is likely, even with maximal dosing. Microbiological resistance is defined by the presence of an acquired or mutational resistance mechanism to the drug in question, in comparison with a fully-susceptible “wild-type”, and may be assessed by genetic analysis or phenotypic testing against a wild-type cut-off value. The clinical resistance scenario is clearly not applicable in the case of biocides, so to avoid ambiguity it is desirable to avoid using “resistance” in relation to the activity of biocides [25]. Similarly, the non-specific use of the term “tolerance” is discouraged, in deference to its technical meaning of an increase in the minimum bactericidal concentration of a substance without alteration in the MIC [55]. The preferred terminology of many authors concerning variation in the effects of biocides upon bacteria is “reduced/increased susceptibility”, or variants thereof. This has been adopted in the present review.

The testing of antibiotic susceptibility by determination of MIC has a sound basis in clinical application, as lethality and speed of killing is less significant than suppression of growth, in the face of immune system activity. However, for biocides the time required for complete killing of the target organisms, commonly measured as a ≥5 log reduction in viable count [56], is in most cases a more appropriate measure of effectiveness, and a prolongation of this kill time may be viewed as good evidence for reduced susceptibility. For some biocides, such as QAC and chlorhexidine, there appears to be a reasonably consistent relationship between changes in MIC and in kill time against planktonic suspensions of certain bacteria [57], but for many others correlations between MIC and kill times are poor or not seen [25,43,57,58,59].

Additional major complications are the substantial differences between kill tests conducted in suspension and on surfaces, and with organisms in differing states of growth, attachment and water stress [25,26,39,56,60]. Indeed, there are few “standard” biocide tests that attempt to mimic accurately and repeatably the likely in-use conditions, and much biocide research is conducted using MIC or minimum bactericidal concentration (MBC) tests in suspension or on solid microbiological media. These have the merit of being inexpensive and repeatable, and they lend themselves to mass screening of many compounds, formulations, bacterial strains and conditions. However, putative reductions in susceptibility identified through such tests may prove not to have significant effects on biocide kill times in more realistic tests of activity [25,39].

Nonetheless, where evidence of co-resistance is being sought, MIC testing can reveal significant alterations in antibiotic susceptibilities even though MIC values for biocides need to be interpreted with caution.

In terminology to accurately describe linked changes in susceptibility among antimicrobial compounds (co-selection) there is an important distinction between cross-resistance and co-resistance [39,61,62]. Links arising as a consequence of physiological adaptations that have effects on the action of a number of agents, are termed cross-resistance. Examples include efflux pump upregulation or over-expression, reduced cell envelope permeability, or (usually in relation to antibiotics) alteration in a target site or acquisition of a neutralising enzyme (such as an extended-spectrum β-lactamase) that prevents the action of agents from more than one class. By contrast, where the mechanisms of reduced susceptibility are dissimilar but are selected together owing to genetic linkage, the phenomenon is termed co-resistance. There is another common definition of cross-resistance, *i.e.*, resistance to related compounds such as members of the same antibiotic family, but this is too narrow to be of much use in the present discussion.

### 3.2. Identified Mechanisms of Co-Selection

On initial consideration, differences between antibiotics and biocides in respect of targets and of modes and intensity of action, would suggest that there is not likely to be much common ground between the two classes of agents in respect of reduced susceptibilities of micro-organisms. Indeed, antibiotic effects on bacteria can be markedly reduced by single-step mutations in target enzymes, or by the acquisition of neutralising enzymes such as β-lactamases [34]. Clinical doses are carefully controlled to avoid toxicity or other detrimental effects and distribution of antibiotics into certain tissues may be poor. Therefore such specific, single-step changes can result in clinical resistance to antibiotics, or to further selection for higher resistance. Similar mechanisms are seen only rarely against biocides (mutation and upregulation of *fabI* in relation to triclosan [42] being a case), and are considered likely to have little effect in “real world” applications where application concentrations are typically multiples of the lethal dose and several targets are affected simultaneously [25].

However, there are some phenomena that confer reduced susceptibility both to biocides and to antibiotics and which are either part of the normal adaptive repertoire of micro-organisms (intrinsic) or are readily acquired by mutation or genetic transfer under appropriate conditions. Intrinsic resistance may be specific to a bacterial grouping (such as mycobacteria or spore-formers), may be exhibited following phenotypic adaptations to the environment (such as biofilm formation or nutrient stress responses), or be associated with survival in relatively protected environments, such as phagocytes in the body or protozoa in the soil [25,26]. The phenomena of low cell wall permeability and active efflux of toxic molecules by multi-drug transporters, discussed below under adaptive resistance, can be considered part of intrinsic resistance mechanisms in some instances, notably in the case of *Pseudomonas aeruginosa*, which exhibits resistance to many antibiotics by virtue of small outer membrane porin channels and vigorous efflux activity [26]. Whilst some of these examples may manifest as apparent reduced susceptibility to antibiotic or biocide without there being any long-term change to the organism, they do potentially allow some proportion of the bacterial population to survive an otherwise terminal challenge, increasing the risk of selection of organisms permanently adapted to the antimicrobial agent.

By contrast to intrinsic resistance, acquired or adaptive resistance is associated with changes in the organism caused by mutation and/or altered expression of endogenous genes, or by transfer of mobile exogenous genetic material [2,63]. Adaptation may be a constitutional and essentially non-reversible effect, typically following mutation. Alternatively, it may be a transient and regulated response to the conditions. Triclosan appears to select for mutational adaptation in *Stenotrophomonas maltophilia* more intensely than does the QAC benzalkonium chloride [64], indicating that biocides differ in their propensity to induce mutational adaptation in any given organism. Some mechanisms can manifest as either stable and constitutional or transient and regulated types of adaptation, with the Multiple Antibiotic Resistance (MAR) phenomenon of *Enterobacteriaceae* (discussed later in this section) being a case in point. The acquisition of resistance mechanisms by genetic transfer may, depending on the case, present a more or less regulated adaptation. Acquired and adaptive mechanisms associated variously with co- and/or cross-resistance include: biofilm capability, multi drug efflux, altered cell envelope permeability, and target site mutation and over-expression.

Biofilms have an extracellular matrix that provides a diffusion barrier and an enhanced medium for bacterial signalling and genetic exchange, plus a potential site for neutralisation or binding of chemical agents: enzymes degrading triclosan, chlorhexidine and some oxidising agents, among others, are known [2,12,65]. Biofilms similarly provide an extracellular site for sequestration of metal ions [6,52]. Minor elevations in MIC may have increased significance for survival of a bacterium in the context of a biofilm diffusion gradient [66]; furthermore, bacteria in the centre of biofilms are relatively nutrient-deprived and therefore may exhibit stress responses that additionally diminish their susceptibility to chemical attack [38,65]. There is evidence for protection of both Gram-positive and Gram-negative bacteria by biofilm, and elevated MIC values in the order of 10 to 1000-fold have been observed for benzalkonium chloride and triclosan, among other biocides, in respect of activity against biofilmed *versus* planktonic bacteria including *Listeria* spp. [2,12]. Biofilm formation is likely to be part of the normal environment-responsive repertoire for capable organisms, although in some cases there may be selection of mutants with hyper-expressed biofilm capacity [67,68].

Active transport of molecules to the periplasmic or extracellular environment via multi-drug efflux pumps is a well-established feature of biocide and antibiotic resistance [69], either as intrinsic or acquired mechanisms. Many Gram-negative organisms encode chromosomal broad-substrate efflux pumps [70], and a variety of multi-drug pumps are similarly-encoded by some Gram-positive organisms including *S. aureus* [69,71]. Alternatively, acquired resistance may be associated with efflux pumps introduced on mobile genetic elements among both Gram-positive and Gram-negative bacteria, or with mutations causing constitutional over-expression of Gram-negative efflux activity [26].

Multi-drug efflux pumps actively expel a broad range of unrelated and structurally diverse compounds, which includes major biocide classes, dyes and antibiotics, driven by trans-membrane ion gradients (protons or sodium) or ATP hydrolysis. Poole [34,69] and Putman *et al.* [72] have provided excellent reviews. Substrates characteristically have a high degree of hydrophobicity, and there is fair evidence that, in many cases at least, the cytoplasmic membrane, rather than the cytoplasm, is the site of initial binding of compounds [72]. This may contribute to the effectiveness of efflux pumps against primarily membrane-active biocides. Whilst efflux pumps may provide only modest reductions in susceptibility to antimicrobial compounds, they appear to have an additional role in the development of higher-level resistance, either by additive or synergistic action with other mechanisms [73], or by increasing the frequency of mutational high-level resistance by allowing a greater proportion of organisms to survive antimicrobial exposure [69].

There are also efflux pumps that expel heavy metal ions, such as elements of the *czc* system encoding a pump for zinc, cobalt and cadmium, and *pcoA*, part of a copper extrusion system [45]. Whereas some efflux pumps for heavy metals may not have broad substrate affinities, there is evidence of co-transport of non-metals in other cases [52].

Expression of efflux pumps is typically under transcriptional regulation by DNA-binding products of activator and repressor genes [72]. These may be products of the particular efflux pump gene cluster or of a global regulator. The AcrR repressor and MarA activator of the AcrAB-TolC efflux pump of *E. coli* and *Salmonella* spp. are local and global regulatory products, respectively. Plasmid-borne efflux pumps, such as the QacAB QAC exporter of *S. aureus*, appear to encode similar regulatory apparatus in addition to structural genes [74]. Transcriptional regulators may themselves be upregulated by substrates or bind them directly, thereby providing routes by which efflux activity may be modulated by the presence of potentially toxic substances [72]. This protects the cell from expending energy on indiscriminate extrusion of metabolically useful compounds when toxic substrates are not present.

Efflux pumps may be upregulated by biocides, for example chlorhexidine or benzalkonium chloride acting on the MexCD-OprJ pump of *P. aeruginosa* [69] or QAC and other lipophilic cations acting on the QacR repressor [74]. Thus, strains possessing such efflux pumps and adapted to survive challenge by biocides that upregulate them may have developed a hyper-expressing phenotype that is nonetheless still repressed in the absence of the biocide. However, many substrates do not stimulate their own efflux and this applies to some biocides, for example triclosan in the case of AcrAB-TolC pump. In this scenario, a bacterial strain with efflux-mediated reduced susceptibility to a non-upregulating biocide will likely be a constitutively-expressing mutant, selected from its un-mutated and therefore fully-susceptible peers, and will not therefore downregulate the efflux once the biocide exposure has waned [75]. This potentially makes such strains a more persistent risk for cross-resistance, although there will be a fitness cost in the absence of selective pressure. Sometimes, increased efflux may paradoxically result in increased susceptibility to an antimicrobial substance [72], possibly as a result of the extrusion of protective substances.

Alterations in cell envelope permeability have substantial potential for cross-resistance, as either intrinsic or adaptive phenomena. Recognised changes associated with reduced susceptibility to biocides relate to the outer membrane of Gram-negative organisms, namely altered fatty acid and protein profiles, and changed surface hydrophobicity and ultrastructure [3,12,44]. However, biocide-adapted bacteria with altered outer membrane proteins do not necessarily show altered permeability in assays [76]. For primarily membrane-active biocides, such as QAC, changes in the cell envelope might also constitute a target site alteration, in addition to a permeability barrier. However, the cytoplasmic membrane appears to be a primary target for QAC and changes here have been little investigated.

Examinations of the role of the cell wall in respect of biocide resistance have also been limited. The waxy, hydrophobic nature of the mycobacterial wall, coupled with narrow cell envelope porins, is associated with resistance of this genus to many hydrophilic biocides [2,12,77]. Using the fractional distribution of cell suspensions in aqueous *vs.* xylene phases Alexander *et al.* [21] reported consistent and significant changes in cell surface hydrophobicity between disinfectant-susceptible and non-disinfectant-susceptible strains of both Gram-positive (*S. aureus*) and Gram-negative bacteria. Similarly consistent changes were also noted following higher *versus* lower challenge concentrations of disinfectant.

It is perhaps unsurprising that some of the above mechanisms may be found operating in concert. For example, many cases of reduced susceptibility to antibiotics and/or biocides show both increased efflux and cell permeability changes [39,40]. Global regulatory responses may drive cross-resistance, by a combination of the mechanisms already described and probably other changes associated with protection and repair of the biosynthetic and metabolic machinery of the cell [78]. These changes may be reversible stress responses or mutational shifts. A well-studied example of the latter is the MAR phenomenon in *E. coli* and other members of the *Enterobacteriaceae*. This is a common phenotype associated with antibiotic resistance or controlled exposure to biocides [24]. On its own it generates modest increases in MIC values (around four- to eight-fold) for a range of antibiotics [12]. Sensitivity to organic solvents, triclosan and pine oil are characteristically decreased, but effects on other biocide MIC values vary according to the particular biocide or biocide mix, and there may indeed be no significant change [78]. Affected systems are under the influence of the *marRAB* operon, and the phenomenon arises following mutational or substrate-mediated inactivation of the MarR transcriptional repressor, leading to increased expression of the MarA global regulator and causing, among other things, upregulation of the *mar* operon itself plus the AcrAB-TolC multi-drug efflux pump, and downregulation of the OmpF porin [72,79,80]. Substrates that may reversibly activate the *mar* regulon include salicylate, bile salts and the antibiotics tetracycline and chloramphenicol. It appears that a principal mechanism mediating reversible MarR inactivation in *E. coli*, and probably other *Enterobacteriaceae* at least, is liberation of copper from the cell envelope under the action of stressors, with subsequent interaction between copper (II) ions and MarR [81]. Whether this effect is seen under direct copper stress remains to be investigated.

It is clear that other global regulatory systems may exert similar effects, generating cross-resistance in the face of external stimuli. The SoxS (oxidative stress) and Rob regulators in *E. coli* are homologues of MarA, activating the *mar* operon as well as other systems [24,38,72]. Furthermore, inter-bacterial signalling may promote cross-resistance in whole populations. Secretion of tryptophanase by relatively resistant *E. coli* subjected in a bioreactor to fluoroquinolone or gentamicin was associated with increased efflux, other stress responses and increased fluoroquinolone MIC values among other strains in the same reactor [82]. This suggests that the spontaneous or regulated development of resistance to antimicrobial challenge by some members of a bacterial community may, via signalling, increase the likelihood of survival of other members of that community by mechanisms common to other cross-resistance phenomena. Potentially this may allow longer survival or more survivors, in turn increasing the likelihood of the development of higher-level resistance.

Co-resistance, arising from the selection of linked genes encoding otherwise unrelated resistance mechanisms to different antimicrobials, is a well-recognised phenomenon in antibiotic use and resistance, such as in the case of the chromosomally-encoded penta-resistance typical of *Salmonella* Typhimurium DT104 [83]. However, co-selection via such genetic linkages can occur more broadly, across genes affecting susceptibility to biocides, heavy metals and antibiotics. Co-location of such genes on mobile elements, such as plasmids and transposons [25,84], also raises the possibility of transfer of co-resistance between bacteria. An obvious example is provided by class 1 integrons, which encode a QAC efflux mechanism (*qacEΔ1*) plus sulphonamide resistance (*sul1*) and variable other antibiotic resistance genes [85]. However, the true significance of QAC efflux in class 1 integrons is debatable, not least because QacEΔ1 is a functionally attenuated Qac pump variant [86]. Variations between bacterial genera in the existence of resistance-related genes and their linkage in the same organism make co-resistance a variable and somewhat arbitrary phenomenon [74,87].

### 3.3. Effects on Bacterial Fitness

Adaptation for reduced susceptibility to antimicrobial substances often comes with associated costs to the organism. This is particularly significant when the change is mutational resulting in constitutive expression of the resistance mechanism [26]. In the present context, broad substrate efflux pumps provide a good example as they consume cell energy resources and indiscriminately remove some useful metabolic substances from the cell. When a non-upregulating biocide selects for a strain that over-expresses efflux constitutively (such as mutants in MepR or MarR repressors of *S. aureus* or *E. coli*, respectively), this strain will subsequently incur the costs of efflux regardless of the presence of the biocide [25,71,72]. Plasmids encoding resistance to biocides or heavy metals plus antibiotics provide another example, where instability and the fitness cost require a selective pressure for their maintenance [88]. However, in this latter example, the minimum selective concentration of the agent may be very low in comparison with its MIC [88].

There may be immediate evidence of fitness costs in the laboratory, in the form of reduced colony size or other growth characteristics [42,76,88], or the cost may be more subtle and seen only in communities where there is competition within and between microbial species. However, co-selection is not uniformly costly and extended exposure may select for compensatory adaptations to restore fitness [38]. Furthermore, some resistance adaptations, such as biofilm enhancement, may enhance survival in other environments [68].

### 3.4. Other Related Phenomena

It is recognised that antibiotic exposure has a non-specific effect on the frequency of mutation and of horizontal gene transfer among bacteria, which may not be associated with any change in susceptibility to the inciting antibiotic [13]. Similarly, the frequency of mutation to antibiotic resistance among *Salmonella* spp. is elevated following exposure to sub-inhibitory concentrations of phenolic biocides or triclosan [89]. Non-specific mutagenesis may be a factor in instances where biocide exposure leads to decreased sensitivity to antibiotics but an unchanged, or even reduced, ability to cope with the biocide itself [44,90]. Furthermore, effects (positive or negative) have been noted with biocides at sub-lethal concentrations in respect of plasmid and phage transfer frequencies [25,26,91].

Susceptibility to metals may be reduced by complexing or sequestering the metal ions or by chemical reduction to less toxic states [6,52,92]. Furthermore, there are several identified interactions between antibiotics (or bacterial resistance to antibiotics) and heavy metals, which may have co-selective or counter-selective consequences. These include: formation of complexes between metal cations and certain antibiotics (for example tetracycline) leading to altered absorption and distribution, degrading of β-lactam antibiotics by copper, and metal dependence of certain resistance mechanisms such as β-lactamases and some tetracycline efflux [93,94,95].

In order for selection of biocide-insusceptible strains to occur, some proportion of the bacterial population must survive application of biocides, and this is one scenario where the mode of use of biocides would appear to offer few opportunities for survivor selection, compared with antibiotics. However, microbes in stressed or biofilm communities will often show intrinsic resistance and survive a severe challenge [26,62,96], as discussed above. Furthermore, use of biocides in the presence of heavy organic soiling or with diluting water containing interfering organic or mineral substances, such as may occur on farms, can be associated with marked reductions in efficacy even at recommended application concentrations [12,97]. An allied phenomenon, again of particular relevance to farms but also to sewage treatment and disposal, is the relative environmental persistence of many biocides such as QAC and triclosan and their tendency to bind to organic matter and soil [39,70]. This provides a potential long-lasting low-level exposure of microbes to selective pressure. Concentrations of triclosan in biosolids and sediments have been reported to lie between the MIC of “fully-susceptible” and “tolerant” bacteria [41].

When considering the effects of biocides and heavy metals on communities of bacteria, changes in the structure and diversity of the community become important elements, such as a shift to predominantly intrinsically-resistant Gram-negative bacteria in the genital tract of catheterised patients subjected to repeated antiseptic washes [22]. Work with sub-therapeutic antibiotic pressures on pig gut communities *in vivo* have shown substantial variability in observed effects, with changes in some cases appearing to be minor possibly owing to shifts occurring predominantly at strain or species-level [98]. Community-wide changes are often examined in the context of water, soils, sewage and other biosolids, where sub-lethal concentrations of antimicrobials are typical. Phenotypic testing (using cultural and non-cultural methods) and metagenomic approaches allow examination of community-wide antimicrobial sensitivities and the frequency of known antimicrobial resistance genes [47,99,100]. Such studies tend to focus on the effect of a known selection pressure on the frequency of a number of resistance genes, on genetic transfer rates and on sensitivity of the bacterial community to other antimicrobials. The risks of co-resistance arising in specific pathogenic bacteria are considered in relation to shifts in this background.

## 4. Co-Resistance in Practice: Observational and Experimental Evidence

### 4.1. Biocides

Many surveys and investigations have involved hospitals or other healthcare environments and *S. aureus*, particularly the β-lactam-insusceptible meticillin-resistant *S. aureus* (MRSA) has commonly been targeted. *S. aureus* strains encode a variety of chromosomal and plasmid-borne efflux pumps, and laboratory chromosomal efflux mutants to single-agent biocides show co-selection for reduced fluoroquinolone sensitivity [71]. However, findings in respect of *S. aureus* biocide-antibiotic co-resistance have in general been highly variable between studies, between strains and between biocides [3,26,43,57,69,101], no doubt affected to some extent by differing methodologies. Apparent biocide tolerance may not be stable and associations seen when comparing MIC values may not be evident when considering kill rates [57], whilst interpretation can be further hampered by a lack of control isolates to validate an observed specific association.

Other investigations of field strains from healthcare sources have yielded similarly variable findings. An early study examined Gram-negative isolates from urine infections submitted by hospitals or community practitioners and reported MIC values to be above in-use concentrations of some cationic biocides, including chlorhexidine and cetrimide (QAC), among certain genera (*Pseudomonas*, *Proteus*, *Providencia*) associated with multiple antibiotic resistance [22]. Hospital strains of *S. aureus* (considered cloxacillin-resistant) and Gram-negative bacteria (gentamicin-resistant) commonly exhibited MIC values for chlorhexidine, cetrimide and chloroxylenol (a phenolic disinfectant) that were above their respective in-use concentrations, whereas corresponding antibiotic-sensitive strains were generally more susceptible [21].

By contrast, and using surface disinfection tests, hospital-derived antibiotic-resistant strains of staphylococci and Gram-negative pathogens did not show significant or consistent differences in kill rates using QAC or phenolic disinfectants, when compared with antibiotic-sensitive laboratory strains [96]. Vancomycin-resistant and -sensitive enterococci showed no differences in MIC values for QAC and chlorhexidine antiseptics [57].

A prospective investigation examined *E. coli* isolates from bacteraemias in one hospital and found no correlation between source or eventual mortality and the MIC for two QAC compounds [102]. An association was observed in this study between MIC of the QACs and a trimethoprim-sulphonamide systemic antimicrobial (co-trimoxazole) but QAC MIC values remained below 1% of in-use concentrations. A follow-up integron analysis confirmed the presence of class I integrons, characteristically encoding a QAC efflux pump (*qacEΔ1*) and sulphamethoxazole resistance (*sul1*), plus in this case trimethoprim resistance [85,103].

Several surveys have been performed on biocide (plus or minus antibiotic) susceptibility patterns in bacterial isolates from livestock units. In a study of environmental bacteria recovered from hatcheries in the USA, non-susceptibility to in-use concentrations of QAC, phenolic and glutaraldehyde biocide products was observed among 19 of 350 isolates, these being low-pathogenic Gram-positive and Gram-negative species [104]. Glutaraldehyde was the most commonly tolerated biocide, which correlated with the frequent and long-term use of this agent in hatcheries. Among 569 Danish livestock isolates examined for MIC in respect of a panel of biocides, no distinct subsets of “low-susceptibility” bacteria were seen, although the MIC ranges were rather wide in some cases: 128-fold and 512-fold ranges for chlorhexidine and benzalkonium chloride, respectively [53]. Using 310 Gram-positive isolates from milking cow teats that had been regularly subjected to iodine or chlorhexidine antisepsis, Martin and Maris [105] demonstrated a significant association among streptococci between reduced susceptibility (elevated MIC) to chlorhexidine and to ampicillin, tetracycline and three aminoglycoside antibiotics. However, no association was seen between bacteria showing elevated MIC values to chlorhexidine and its use on source animals.

Biocide-manufacturing plants are other environments with bacteria that potentially are regularly exposed to biocides. Isolates from plants manufacturing the phenolic agents triclosan or chloroxylenol were typically of low susceptibility to these agents, or from genera with intrinsically low susceptibility such as *Pseudomonas* [106]. For those isolates with elevated MIC values to triclosan or chloroxylenol there was no evidence of associated reductions in susceptibilities to other biocides (chlorhexidine, benzalkonium chloride or phenol), nor to any of a panel of antibiotics [59].

Laboratory investigations into co-selection often include “training” of field or laboratory strains by repeated subculture with stepwise increases in the concentration of biocides in liquid or solid media, by the use of biocide concentration gradients in solid media, or by extended culture in biocides at near-inhibitory concentrations. This may yield strains with decreased susceptibility to the “training” biocide and other antimicrobials (usually in terms of increased MIC), which may or may not be stable upon subculture in the absence of the biocide. Alternatively, there may be little or no change in MIC value for the training biocide, but more marked changes in those for certain antibiotics. Findings tend to be highly individual to the particular combination of biocide and bacterial strain being examined [25]. Whilst being a useful tool for investigating possible mechanisms of co-selection, the relevance to “real world” interactions of such carefully-controlled and prolonged exposure to biocides has often been questioned.

Stepwise training procedures for triclosan may produce dramatic increases in MIC (three or four orders of magnitude) for *E. coli* and some *Klebsiella* isolates, whereas changes reported for other species including staphylococcal human skin isolates are far smaller, in the order of one- to ten-fold [68,107,108,109,110]. Data for training *Salmonella enterica* serovars with triclosan is inconsistent, with minor effects in one report [108], and MIC values elevated 64- to >512-fold in others [42,111]. For other commonly-tested single-agent biocides such as chlorhexidine, chlorine, peroxyacetic acid and QAC including benzalkonium chloride, training procedures tend to yield up to eight-fold elevations in MIC, with occasionally a decrease in MIC being observed for certain combinations of bacterial strain and biocide [67,76,109,112,113].

Following such biocide training of strains, allied shifts in susceptibility to other biocides and antibiotics are commonly reported, although variable in nature [41]. Bradoukai and Hilton [107] reported reductions in susceptibility to a panel of antibiotics and to the other test biocides, following stepwise training of *E. coli* O157 and various *Salmonella* serovars against triclosan, chlorhexidine and benzalkonium chloride. The authors reported shifts occurring in a minority of strain-antimicrobial pairings, with the pattern varying between species and between *Salmonella* serovars. Increases in antibiotic MIC values in this study varied between two- and 500-fold. Exposure training of veterinary field isolates and a laboratory strain of *E. coli* to three QAC compounds yielded elevations of MIC that were in some cases above clinical breakpoints for resistance, for a number of antibiotics: phenicol, tetracycline, fluoroquinolone, β-lactam classes and trimethoprim [113]. Stepwise training of *E. coli* strains with benzalkonium chloride or chloramphenicol resulted in mutual increases in MIC: around six-fold for the QAC training agent, two-fold for the QAC after antibiotic adaptation and over twenty-fold for chloramphenicol after the QAC adaptation [76]. Benzalkonium chloride-adapted strains showed higher survival in lethality tests and increased MIC values, of 1.5- to >20-fold, for antibiotics in most cases.

Training *Listeria monocytogenes* and *Salmonella* serovars Enteritidis and Typhimurium with five poultry carcase decontaminants (including chlorine dioxide, acidified sodium chlorite and peroxyacetic acid) resulted in reduction in antibiotic susceptibilities to therapeutic compounds in a minority of cases, past clinical resistance breakpoints in 13 of 300 pairings [112]. There was no strong pattern, although development of resistance to the aminoglycosides streptomycin and neomycin was common. By contrast, triclosan training of a number of *Salmonella enterica* serovars was associated with neutral effects on sensitivity or, in the case of the two aminoglycosides tested (gentamicin and kanamycin), increases in susceptibility [42]. Reviewing the evidence on peroxyacetic acid, chlorine, chlorite and trisodium phosphate as a poultry carcass and meat decontaminant, the European Food Standards Agency concluded that there was no evidence that their use would lead to acquired reduced susceptibility among contaminating bacteria, nor to acquired resistance to antibiotics [114].

Shorter single exposures to sub-lethal concentrations of biocides appear to have effects upon antibiotic sensitivity that are similar or related to those observed with stepwise training procedures. *Salmonella* Enteritidis cells surviving a short exposure to in-use concentrations of chlorine exhibited up to eight-fold increases in MIC values for tetracycline, nalidixic acid and chloramphenicol [115]. When the low-pathogenic Gram-negative anaerobe *Bacteroides fragilis* was exposed to triclosan, benzalkonium chloride, oxidising agents and chlorhexidine only the last was associated with elevated MIC values to antibiotics, these being of the β-lactam and tetracycline classes but not chloramphenicol or a fluoroquinolone [116]. Interestingly, non-biocidal drugs known to induce efflux (salicylate, ibuprofen, paracetamol) exerted a similar effect as chlorhexidine. Similarly, one-time exposure of two *E. coli* strains to benzalkonium chloride, salicylate, a bile salt and the oxidising stressor methyl viologen resulted in modest increases in MIC values for benzalkonium chloride and chloramphenicol in most cases [76].

In some examinations of biocide training and the co-selection of antibiotic resistance, a role for efflux can be demonstrated or reasonably inferred. Inactivation of the AcrAB-TolC efflux pump increased *E. coli* susceptibility to triclosan ten-fold, whilst overexpression of AcrAB-TolC or induction of the MAR phenotype increased the triclosan MIC by factors of 3 and 20, respectively [117]. Among benzalkonium chloride-adapted *E. coli*, ethidium bromide efflux was increased for one of two strains in the study by Langsrud *et al.* [76]. Partial or complete reversal of acquired antibiotic resistances upon application of an efflux pump inhibitor have also been reported in *E. coli* by Soumet *et al.* [113] and in *B. fragilis* by Pumbwe *et al.* [116].

Reduced susceptibility of *Salmonella enterica* serovars to triclosan was partially reversed by exposure to an efflux pump inhibitor [42], whilst three *Salmonella* serovars with reduced susceptibilities to benzalkonium chloride, triclosan or erythromycin following training showed a return to susceptibility in the presence of an efflux inhibitor [118]. Over-expression of AcrAB was mildly protective of *Salmonella* against some biocides, but not an oxidising mix [119], whilst introducing the MAR repressor (MarR) on a plasmid abolished increases in antibiotic MIC associated with chlorine exposure in *Salmonella* Enteritidis [115].

Evidence for efflux in co-selection comes from other sources also. Multidrug efflux pump genes were far more common than antibiotic-specific resistance genes among Gram-negative food isolates with reduced susceptibility to biocides plus antibiotics [120]. If co-selection had occurred in these bacteria, then the dominant mechanism appeared to be cross-resistance, probably involving efflux.

Where cross-resistance mechanisms are involved, reduced susceptibility to biocides may follow from the development of antibiotic resistance, as well as vice versa. Reciprocal, albeit asymmetric, adaptation to benzalkonium chloride and chloramphenicol by *E. coli* reported by Langsrud *et al.* [76] provides a good example, as does an investigation whereby fluoroquinolone- or tetracycline-resistant *E. coli* or *Salmonella* strains inoculated onto cloths were killed less efficiently by phenolic biocides than were antibiotic-susceptible strains [121]. Evidence for efflux activity was presented.

Multi-drug efflux mechanisms often are plasmid-borne among Gram-positive bacteria [26], and mobile efflux pumps are seen among Gram-negative organisms too. Where efflux genes on mobile elements are accompanied by specific antibiotic resistance genes [122], cross-resistance and co-resistance may be acquired together. Clinical isolates of *E. coli* with reduced QAC and antibiotic susceptibilities had integrons encoding both the QacEΔ1 efflux pump and trimethoprim/sulphonamide resistance, and in addition over-expressed the AcrAB-TolC efflux pump [103]. The Qac efflux pumps are widespread among staphylococci and a range of Gram-negative bacteria, being typically plasmid-borne and often occurring in conjunction with specific antibiotic resistance genes [12,39,74]. Although Qac-type pumps will expel a number of lipophilic cationic compounds including QAC and other biocides, evidence for their significance in biocide insusceptibility is conflicting [74,123], and their association with antibiotic resistance may owe more to genetic linkage with resistance genes than to efflux-mediated cross-resistance.

In a study specifically examining biofilm-forming capability, *E. coli* isolates from dairy equipment that had reduced susceptibility to benzalkonium chloride and/ or ciprofloxacin proved to have superior biofilm capacity, whilst training more susceptible isolates with the antimicrobial agents led to decreased susceptibility, of varying stability, in parallel with improved biofilm formation and evidence of increased efflux activity [67]. Improved biofilm capability plus efflux has also been seen with triclosan-adapted *E. coli* [68], and *Salmonella* Typhimurium in biofilm were substantially more resistant to killing than were planktonic stationary-phase cells [60].

Using a bacterial community approach to examining co-selection, bioreactors were seeded with river sediment communities and subjected over four years to varying nutrient and benzalkonium chloride concentrations. When exposed to the QAC, community diversity reduced and community-wide MIC values for benzalkonium chloride, ciprofloxacin, tetracycline and penicillin G all increased [100]. The frequency of efflux genes increased in biocide-exposed populations and an efflux inhibitor reduced the antibiotic MIC values, but interestingly not that of the QAC. Thus, population restructuring, genetic transfer, efflux and other resistance mechanisms were all potentially involved in this model microcosm of long-term sediment contamination by QAC.

### 4.2. Heavy Metals and Other Agents Added to Animal Feed

Co-selection mechanisms impacting antibiotic susceptibility have been identified for heavy metals that are similar to those for biocides, namely cross-resistance via extracellular adaptations (biofilms especially), cell envelope changes, efflux and global regulatory responses, plus co-resistance via gene linkage [6]. Important respects in which heavy metals differ from biocides include their mode of use in agriculture and aquaculture, where they are used either continuously or for an extended period of time and usually at sub-lethal concentrations, and their marked tendency to persist in manure and the environment [6]. Indeed, the extended relationship between pathogens and copper in agricultural waste, which may then be mixed with the soil microbiota, has prompted suggestions that this may be an especially advantageous scenario for the development and transfer of multiple resistance determinants in pathogenic bacteria [6,47]. By contrast, some heavy metals (particularly mercury and lead) have been associated with counter-selection leading to reduced antibiotic susceptibility, probably via a variety of mechanisms [6,94].

Many metal-antibiotic insusceptibility correlations have been observed in samples retrieved from heavy metal-contaminated environments, and there are identified cross-resistance mechanisms that can effect such changes including efflux (*Listeria monocytogenes*, *Pseudomonas aeruginosa* and *E. coli*) and co-regulation, via *robA* in *E. coli* and *czcS* in *Pseudomonas aeruginosa* [52]. In soil long-contaminated by historical industrial use of copper and without recent agricultural activity, both culture and non-cultural viability assays of soil bacteria showed decreased copper and antibiotic susceptibilities, compared with local matched control samples [47].

Despite much data on correlations, evidence for causal links remains scant [6]. An exception is provided by Stepanauskas *et al.* [99] who reported that river water microcosms subjected to heavy metal (nickel or cadmium) stresses yielded significantly increased frequencies of bacteria resistant to antibiotics, and vice-versa. Similarly, copper amendment of soil to concentrations near the European Union limit for sewage-treated soils was followed by a shift towards reduced copper susceptibility in cultured organisms from the soil microbiota and an allied decrease in multiple antibiotic susceptibilities, close to clinical breakpoints in many instances [124]. In the latter study, there was an associated change towards Gram-negative isolates, and many such metal-antibiotic susceptibility correlations may reflect a shift in bacterial community structure and diversity towards naturally-resistant organisms, with limited implications for animal and human pathogens. However, there is evidence of co-resistance in other cases [6]. A relevant example is the recently-reported linkage between *czrC*, associated with reduced zinc and cadmium susceptibility, and the SCCmec genomic island characterising human and livestock-associated MRSA strains [125]. The copy number of tetracycline- and sulphonamide-resistance genes (*tetA*, *sul1*) in DNA extracts from weaner pigs was significantly increased in animals heavily supplemented with zinc [126], although to what extent that reflected selection of insusceptible organisms *vs.* a community shift is unclear.

Homeostasis of metabolically-important metals may rely on chromosomally-encoded uptake, efflux, chaperoning and detoxification mechanisms, such as is the case with copper [92,127]. However, there is fair evidence that heavy metals in contaminated sediments and water may commonly exceed selective concentrations [6] and resistance to toxic external concentrations of heavy metals appears commonly to require additional plasmid-borne genes [26,52,92,128,129]. Under these circumstances co-resistance may be accompanied by enhanced genetic mobility. An example of plasmid-associated co-resistance with contemporary relevance is the *tcrB* gene associated with reduced copper susceptibility [50], which shares homology with the homeostatic efflux-associated *copB* of *Enterococcus hirae* [129]. In *Enterococcus faecium*, *tcrB* is plasmid-borne and shows linkage with *erm*(B) and *vanA*, encoding resistance to erythromycin and vancomycin, respectively [50,129]. The carriage of *vanA* is believed to have been promoted by the glycopeptide growth promoter avoparcin, which was banned in the 1990s amid concerns about cross-resistance with human vancomycin-resistant enterococci [13,130].

There is some evidence that controlled exposure of bacterial pathogens to essential oils can alter antibiotic susceptibilities, but field studies are lacking [131,132,133]. Compared with heavy metals, phytochemical and organic acid feed additives are short-lived following ingestion and, given their limited application currently, their significance for microbial resistance on farms is likely to be minimal.

## 5. Observations on Specific Livestock-Associated Bacterial Groups

### 5.1. Escherichia coli

Field surveys, although limited in scope, have not revealed strong evidence for links between biocide and antibiotic susceptibilities among *E. coli*. Avian-pathogenic (APEC) strains from egg laying flocks were examined for MIC and MBC of five biocide components: two aldehydes, a QAC and hydrogen peroxide [134]. Despite prevalent resistance to several antibiotics, no subset of strains with reduced biocide susceptibility was evident in this study. McMurray *et al.* [117] reported that examination of 31 antibiotic multi-resistant clinical strains did not reveal common overexpression of AcrAB-TolC. By contrast, 570 isolates from meat in the USA where QAC are commonly used as sanitisers in the food chain, frequently demonstrated elevated MIC values for the four tested QAC [135]. There were also significant associations reported in this study between the presence of some plasmid-borne QAC resistance genes [*sugE(p)*, *qacEΔ1*] and phenotypic antibiotic resistance, consistent with co-resistance. However, the specificity of these associations for QAC-exposed *E. coli* was not tested against control strains.

Field strains resistant to ciprofloxacin, including those with upregulated efflux (*acrAB*) or global responses (*marAB*, *soxRS*) showed no difference compared with ciprofloxacin-sensitive strains in susceptibility to three commercial compound disinfectants in MIC and competition assays, nor conversely did disinfectant-passaged strains show a significantly increased frequency of mutation to reduced sensitivity on 4x MIC ciprofloxacin media [136]. However, the authors reported a decrease in cyclohexane sensitivity, consistent with the MAR phenomenon, among a minority of disinfectant-passaged strains (particularly a phenolic disinfectant) and among these, ciprofloxacin, ampicillin and tetracycline sensitivity typically was reduced. Regardless of changes to cyclohexane or antibiotic sensitivity, disinfectant exposure did not significantly alter the studied strains’ MIC values in respect of the disinfectants themselves.

Other laboratory investigations have also emphasised the variable nature of biocide adaptation and of allied effects in respect of antibiotics. Field strains of verocytotoxigenic *E. coli* could be trained to increase MIC values for single-agent biocides (triclosan and benzalkonium chloride) but no stable MIC changes could be achieved for chlorhexidine or commercial biocide mixes, and no increase in antibiotic sensitivities were seen [110]. Adaptation of a laboratory strain to sub-lethal concentrations of food-grade biocides was associated with marked variability, according to biocide, in the degree and stability of reduced biocide susceptibility, changes to biofilm formation, cell membrane hydrophobicity and antibiotic sensitivities [58]. The induction of reduced triclosan susceptibility, and associated increases in antibiotic, chlorhexidine and benzalkonium chloride MIC, occurred to differing degrees and at different rates depending on serogroup [137]. A marked reduction in triclosan susceptibility induced in an O157:H19 strain was associated with mixed effects, including growth inhibition, increased biofilm formation and a non-constitutive triclosan-inducible change in outer membrane proteins [68]. This last study also found that efflux inhibition profoundly increased the sensitivity to triclosan, but only at a high dose of the inhibitor (PAβN), when other toxic effects may have influenced the outcome.

Against a background of prevalent multi-drug antibiotic resistance and moderately low copper sensitivity, administration of copper to weaner pigs in feed at a growth-promotion concentration was associated with significantly increasing susceptibility to most tested antibiotics among faecal *E. coli* [87]. The study also found that copper susceptibility was not decreased, and neither was the frequency of the plasmid-borne copper resistance gene *pcoD* increased. It is possible that baseline levels of antibiotic and copper resistance were sufficiently high in this study population that the copper supplementation was insufficient to select for reduced copper susceptibility or associated antibiotic resistance genes. In a prospective study of faecal *E. coli* among 180 weaners, in-feed copper supplementation at a growth-promoting concentration was associated with variable effects on MIC values of antibiotics over time, with the only significant change compared with control being a reduction of MIC for neomycin and chlortetracycline after six weeks [51]; No significant effects were observed for high concentrations (2000–3000 ppm) of added zinc.

Multivariable modelling of observed copper and zinc concentrations in pig slurry and antibiotic MIC values suggested positive associations between MIC of a minority of antibiotics and one or other of the metals [94]. Negative associations with antibiotic MIC were seen in respect of lead and mercury. Linkage between *pcoD* and the tetracycline resistance determinant *tet*B, but not *tet*A, was observed by Agga *et al* [87], emphasising the often arbitrary nature of co-resistance links. A possible effect on the mobility of antibiotic resistance genes in *E. coli* was observed by Bednorz *et al.* [61], who reported an increased diversity of genotypes and plasmid profiles, and increased multi-drug antibiotic resistance, among pigs supplemented with high levels (2500 mg/kg feed) of zinc.

### 5.2. Salmonella enterica

Among Danish broiler house isolates from the decade up to 2001, MIC values were elevated for certain serovar-biocide combinations but there was no relationship to likely previous field exposure nor was there any temporal change in MIC among strains persistent in an individual broiler house [138]. In this study the MAR phenotype was seen very rarely among those strains with elevated MIC, and triclosan-selected variants showing MAR-type reduced sensitivity to cyclohexane did not show elevated MIC to the original tested biocide products. Over 500 isolates from Danish pig slaughterhouses showed MIC values well below in-use concentrations of a QAC, an acid/hydrogen peroxide mix and triclosan, and post-cleaning and disinfection samples recovered *Salmonella* only from drains [139]. There was little evidence of associations between increased biocide MIC and antibiotic resistance. Field strains from pigs and poultry in Thailand commonly exhibited the MAR phenotype, but this was not associated with reduced susceptibility to benzalkonium chloride or chlorhexidine among 257 strains [140]. Møretrø *et al.* [56] have noted that there is little evidence of resistance by field salmonellae to working concentrations of disinfectants.

Multiple antibiotic resistance was significantly more common among a subset (fewer than 4%) of 428 livestock and human isolates showing modest reductions in triclosan susceptibility [141]. All of the subset in this report showed elevated expression of *acrB* and most also had increased efflux of a fluorescent dye, but mutations in *fabI* were not seen. Similarly, Randall *et al.* [142] reported associated lower susceptibilities to AcrAB-TolC efflux substrates (cyclohexane, ampicillin, tetracycline, ciprofloxacin, a QAC and triclosan), but not to non-transported kanamycin, among isolates from poultry, human and environmental sources.

Several laboratory studies have also supported a substantial role for efflux-associated reduced biocide susceptibility and cross-resistance. Serovars Enteritidis, Typhimurium and Virchow with stable laboratory-induced adaptation (2× to 64× the original MIC) to triclosan or benzalkonium chloride reverted to susceptibility when treated with an efflux pump inhibitor [118]. However, concurrent changes in cell surface charge and hydrophobicity among the mutants suggest other enabling mechanisms were also present.

For *Salmonella* Typhimurium, adaptation to benzalkonium chloride was associated with constitutive overexpression of AcrAB-TolC and, when that was experimentally inactivated by mutation and biocide training repeated, with similar expression of AcrEF-TolC [143]. When the same serovar was subjected to severe biocide challenges (five hours at in-use concentrations) survivors showed consistent reduced growth rates and low-level decreased sensitivity to ciprofloxacin, tetracycline and chloramphenicol, although not to the challenge biocide [119]. The authors reported a MAR phenotype with decreased OmpF and increased AcrEF-TolC-mediated efflux. By contrast, survivors of moderate (five hours in broth at 2× to 4× MIC) challenges showed the same or reduced growth rates as the initial inoculum, and the same survival percentage when re-challenged, suggesting that adaptation under these conditions was largely a reversible physiological process.

Other investigations have emphasised the variability in findings, between strains, biocides and experimental methods. Karatzas *et al.* [111] reported that sequential daily exposure of *S.* Typhimurium to sub-inhibitory concentrations of commercial compound biocides did not yield elevations in MIC to the biocides but, depending on the biocide, stable elevations in some antibiotic resistances were seen, accompanied in some cases by reductions in MIC to the other disinfectants, in growth parameters, and in invasiveness of a cell monolayer. By contrast, Randall *et al.* [144] described single, extended (seven-day) exposure of *S.* Typhimurium to aldehyde, oxidising, phenolic and QAC-based commercial compound biocides at 2× MIC concentrations, yielding strains with reduced susceptibility at frequencies suggesting single-step mutation events. In this study, loss of susceptibility to the biocides was not associated with decreased growth, or with reduced colonisation and shedding from chicks. Additionally, changes in MIC values, proteome expression and biocide kill time varied by strain and by biocide, with kill time for the phenolic biocide actually being shortened among mutants [144].

Some of the mutants in the above report by Karatzas *et al.* [111] showed elevated *acrB* expression, consistent with the MAR phenotype. Of the tested biocides, oxidising and phenolic/tar acid mixes were associated with less reduction of antibiotic sensitivities than QAC and QAC/aldehyde-based products. Follow-up testing of one representative strain for each of three biocide products (QAC/aldehyde, oxidising, phenolic) revealed outer membrane protein changes, decreased motility and invasiveness for all, whereas expression of AcrAB-TolC and changes in lipopolysaccharides varied between biocides [78]. In the contrasting extended single exposure study [144], the MAR phenotype was not observed, but native efflux capability in the form of a functional AcrAB-TolC pump was required for strains with reduced susceptibility to any tested biocide except for the oxidising one. As a final comparison, small-scale *in vivo* competition and transmission trials were performed using oral dosing of chicks with wild-type *S.* Typhimurium and derivatives with the MAR phenotype induced after exposure to oxidising or aldehyde biocides [145]. These showed the MAR mutants to have a fitness disadvantage compared with the parent strain, and to not be preferentially selected by fluoroquinolone treatment.

The variable and sometimes counter-selective effects of biocide exposure were also illustrated by Birošová and Mikulášová [146]. They reported that *S.* Typhimurium, when selected on solid media at between 0.125× and 2× MIC concentration for reduced triclosan susceptibility, showed variable phenotypes in respect of ciprofloxacin, chloramphenicol and tetracycline resistance, depending to some extent on the intensity and frequency of the selection steps. Furthermore, the same study also reported that ciprofloxacin-resistant strains with the MAR phenotype showed no decrease in triclosan susceptibility (and sometimes a decrease in ciprofloxacin resistance) when passaged on 0.5× MIC triclosan media, by contrast to non-MAR equivalent strains where ciprofloxacin resistance was maintained and triclosan susceptibility decreased.

In tests comparing *Salmonella* cells in differing states, six of seven commercial compound biocides at half their in-use concentrations and against planktonic cells were fully lethal for members of a collection of 189 *Salmonella* strains, whilst the remaining biocide was inconsistently lethal for one strain in the collection [62]. Surface-dried cells were less susceptible but generally were still effectively killed at in-use concentrations, whereas cells in biofilm were resistant to killing by several of the products even at full concentration and with extended contact times. Møretrø *et al.* [56] has noted that *Salmonella* in biofilms are unpredictably insusceptible to biocides. Attempts by Condell *et al.* [62] to adapt strains to the compound products were not successful, but stable increases of up to 4× MIC were seen when using individual components (triclosan, benzalkonium chloride, chlorhexidine); these strains did not show reduced susceptibility to the other agents, but did in some cases show decreased sensitivity to some antibiotics.

Carcass and food decontaminant biocides have been examined specifically in relation to *Salmonella*. Exposure of pan-susceptible and multi-drug resistant serovars Typhimurium and Newport (plus *E. coli* O157:H7) to biocides under recommended conditions after being inoculated onto meat showed variations in kill rate that correlated strongly with the particular products and weakly with bacterial species and serovar, but not with the antibiotic resistance phenotype of the strains [147]. Examining the lethality (decimal reduction times) for various serovars of three biocides, Capita [148] reported significant positive associations between antibiotic resistance and resistance to killing by acidified sodium chlorite or trisodium phosphate. Such associations were present in only some of the serovars examined, and no association could be discerned in the case of citric acid. In another study, training multi-drug resistant strains of diverse serovars with trisodium phosphate, sodium nitrate and sodium hypochlorite resulted in modest elevations in MIC values to the training agent [149]. There were also variable effects on antibiotic sensitivity, exceeding clinical resistance breakpoints for a minority (around 10%) of strain-antibiotic combinations (including aminoglycosides, by contrast to efflux mutants discussed previously) and in variable patterns, suggesting that mechanisms were non-specific in respect of antibiotic structures or targets.

Examining heavy metal susceptibility, Medardus *et al.* [45] reported that among 349 *Salmonella* isolates from nine pig units in the USA, an elevated zinc MIC was associated with the occurrence of the *czcD*-encoded zinc efflux pump but not with the concentration of faecal zinc. By contrast faecal copper concentration was associated with an elevated copper MIC, but not with the occurrence of a copper efflux gene *pcoA*. The same study reported that serovars were associated both with copper and zinc susceptibility and with patterns of antibiotic resistance; such resistances were not independently associated with copper or zinc susceptibility once serovar was accounted for.

A Portuguese study of monophasic *S.* Typhimurium variants, mostly of human and pig origin, found that the *sil* operon, encoding an efflux mechanism for copper and silver, was common among the principal clonal groups, and it exhibited an associated phenotypic resistance to the metals, which was restricted to anaerobic conditions in the case of copper [150]. Antibiotic resistance genes in this commonly multi-drug-resistant pathovar were co-located with *sil*, on the chromosome (European clonal group) or a non-transferable plasmid (Spanish and Southern European clonal groups). The authors found that in plasmid-borne cases, many antibiotic resistance genes were located within class I integrons.

### 5.3. Livestock-Associated Meticillin-Resistant Staphylococcus aureus (LA-MRSA)

*S. aureus* is an upper respiratory tract coloniser that is distributed very widely among mammals but which generally shows host-species-specific subtypes [30]. Isolates of clonal complex 398 (CC398) are closely associated with pigs and some other livestock in a number of countries [151], and within the last decade CC398 strains carrying the SCC*mec* genomic island characteristic of MRSA have come to prominence as persistent colonisers of pig herds [30]. These LA-MRSA strains can colonise humans in contact with animals but, unlike community- and healthcare-acquired MRSA, they do not readily spread between people.

A specific association between reduced zinc sensitivity and CC398 MRSA [152] was shown to be a consequence of the frequent presence of *czrC*, encoding a putative metal transporter, in SCC*mec* (type V) of both pig and human isolates [125]. Among weaner pigs, administration of pharmacological in-feed doses of either zinc or tetracycline increased counts on nasal swabs of naturally-occurring *czrC*-positive, tetracycline-resistant MRSA [153,154]. A larger and slightly longer-term study following weaners with pharmacological *vs.* basal in-feed zinc concentrations on a unit with endemic *czrC*-positive MRSA, found a significant association between the prevalence of MRSA-positive animals and zinc level at four and five weeks of age, but in both groups MRSA-positive animals were similarly infrequent by seven weeks of age [155]. A further report by the same group [156] described significant and independent associations between zinc oxide supplementation or frequent disinfection of pens and nasal MRSA in nursery age pigs, on 26 units. Carriage of *qac* genes was universal among MRSA, and reduced susceptibility to benzalkonium chloride was observed amongst a minority of strains. In the same study, *czrC* was detected in about two-thirds of tested isolates, in association with a lower susceptibility to zinc compared with *czrC*-negative isolates.

Other chromosomal resistance determinants for heavy metals (mercury and cadmium) have been associated with meticillin resistance in human MRSA strains [157,158,159]. Moreover, clusters of heavy metal and antibiotic resistance genes have also been found to be plasmid-borne among MRSA strains from humans (ST398, type V SCC*mec* with *czrC*) and pigs (ST398, type IV SCC*mec* without *czrC*) [160]. Genes associated with resistance to macrolides, lincosamides, streptogramin B and tetracyclines [*erm*(T), *tet*(L)] plus, variably, aminoglycosides and trimethoprim (*aadD* and *dfrK*) were co-located with *copA*, *cadDX* and *mco*, encoding copper and cadmium transporters and a copper oxidase. The authors of this study also reported transmissible phenotypic co-resistance to the relevant agents.

### 5.4. Listeria spp.

Resistance to antibiotics, other than intrinsic resistance to many cephalosporins, is uncommon in *Listeria monocytogenes*, the principal species of public health concern [161]. There were no unusual antibiotic resistances identified among 29 strains from a meat processing plant, three of which showed long-term persistence and reduced susceptibility to benzalkonium chloride [162]. Similarly, antibiotic susceptibility was high among 114 food isolates, 8% of which showed modestly elevated MIC values for benzalkonium chloride [163]. Reduced susceptibility to benzalkonium chloride in field strains has been shown to be plasmid-borne and not linked to antibiotic resistance in one study [164] but associated with efflux and small changes in susceptibility to kanamycin in another [165]. In both these studies, efflux-related reduced susceptibility to this QAC plus, variously, ciprofloxacin or kanamycin has been observed in strains subjected to selection with one or other of these agents. Analysis of gene expression in these studies suggested that the efflux system(s) induced by an antimicrobial agent may differ between strains, thus producing differing patterns of co-selection.

Laboratory exposure of *L. monocytogenes* to sub-lethal concentrations of triclosan produced aminoglycoside-resistant mutants that showed no change in triclosan MIC but which exhibited, variously, other phenotypic changes including small colony morphology and increased oxygen sensitivity [90,166]. The same effect was not seen in these studies when QAC or oxidising compounds were substituted for triclosan, but decreased sensitivity to aminoglycosides has been reported elsewhere, alongside reduced susceptibility to benzalkonium chloride following exposure to this agent [165]. The recent publication of the genomes of five *L. monocytogenes* strains with reduced susceptibility to benzalkonium chloride [167] may assist elucidation of the mechanisms involved in co-selection.

### 5.5. Campylobacter spp.

A survey of 42 isolates from human, animal and environmental sources did not show any associations between the (varied) MIC values to single biocides and sensitivities to erythromycin and ciprofloxacin [168]. In another survey, of 443 field strains, modest increases in multiple antibiotic resistances correlated with lowered susceptibilities to efflux substrates (ethidium bromide, acridine orange, triclosan) in a small minority of isolates [169].

A laboratory study training five isolates with five biocides (QACs, chlorhexidine, triclosan and trisodium phosphate) yielded modest, reversible increases in MIC values in 20% of cases, plus reduced MIC in others [44]. The study also reported variable allied decreases in susceptibility to the other tested biocides and antibiotics; investigations suggested that alterations in cell envelope plus efflux mechanisms were involved.

### 5.6. Enterococcus spp.

*Enterococcus faecium and Enterococcus faecalis* are leading causes of hospital-acquired infections, commonly with multiple drug resistances [170]. Schwaiger *et al.* [171] examined both these species from human bacteraemias and faeces plus the latter species additionally from a variety of livestock, meat and dairy sources, in respect of susceptibility to a QAC (DDAC) and to 22 antibiotics. Most isolates with reduced susceptibility to DDAC (compared with wild-type) were from dairy sources, but resistance to “in use” concentrations were not observed in field isolates or laboratory-trained strains. Associations between reduced DDAC susceptibility and resistance to a few antibiotics were observed among human faecal *E. faecium* strains only.

Co-transmission of the copper efflux-associated *tcrB* and erythromycin resistance *erm*(B) genes has been reported between a sediment-derived livestock species (*Enterococcus hirae)* and *E. faecalis* in conjugation experiments [172]. From 569 Danish livestock-derived bacterial isolates tested for susceptibilities to antibiotics, biocides and heavy metals, the only distinct “less susceptible” group of organisms seen were *Enterococcus* spp. in respect of copper sulphate [53]. Furthermore, among pig-derived *E. faecium* and *E. faecalis*, reduced susceptibility to copper and a number of antibiotics was correlated with the intensity of copper supplementation and of antibiotic use in the source countries, and *tcrB* was specifically present in the copper-insusceptible isolates [173]. However, as antibiotic and copper use were themselves closely correlated, the extent of actual co-selection could not be determined. Genomic analysis of *E. faecalis* from copper-supplemented Danish pigs revealed the presence of a chromosomal cluster of copper-insusceptibility genes, including the *tcrYAZB* operon, in three of six isolates [174]. Tetracycline- and vancomycin-resistance genes (*tetM*, *vanA*) were present in all and one of these three “copper-insusceptible” isolates, respectively, whilst only *tetM* was encoded among the “copper-susceptible” strains, in one of the three isolates. The frequency of individual antibiotic resistances among *Enterococcus* spp. from Australian pigs reflected prevalent antibiotic use within the industry, whilst reduced susceptibility to zinc (but not to copper) was common among *E. faecalis* and resistance to vancomycin was present in commensal non-pathogenic species [175].

Among *E. faecium* from Danish farms, *tcrB* was more frequently detected from the more intensively copper-supplemented livestock species, whilst linkage with erythromycin and vancomycin resistance was seen only among (heavily copper-exposed) pig isolates [129]. Copper fed to feedlot cattle at a growth promotion concentration (10× basal requirement) was associated with modestly but significantly increased frequencies of detection of *tcrB*-positive and macrolide-resistant *erm*(B) enterococci with lowered copper susceptibilities, whilst resistances to other screened antibiotics, including vancomycin, were unaffected [176]. A prospective study of weaner and grower pigs given a heterogeneous inoculum of *tcrB*-positive and -negative *E. faecium* reported that exposure at a commercial (versus basal) in-feed concentration of copper was associated with a higher detection frequency of *tcrB* and of the linked *erm*(B) and *vanA* genes [50].

### 5.7. Summary of Data on Specific Bacterial Groups

In general, field surveys have provided weak evidence at best of causal associations between reduced susceptibilities to biocides, heavy metals and antibiotics in these bacterial groups, although data in this area is far from comprehensive. There is experimental evidence that multi-drug efflux is necessary, if not sufficient, in many cases of reduced antibiotic susceptibility associated with exposure to biocides. Furthermore, such efflux-dependence of modestly elevated antibiotic MIC values has also been seen in some field survey isolates although it is not possible to establish clear links with biocide use in these cases. Selection for cross-resistant strains, with perhaps efflux and envelope adaptations, appears plausible for *Enterobacteriaceae*, at least, under field conditions given the survival of cells even under severe challenge in the laboratory. Biofilms have the potential to enhance this substantially.

One overarching theme is the marked variability in results seen between experimental studies, even examining highly similar organisms and biocidal agents. This suggests that several mechanisms may be involved, to a greater or lesser extent, when co-selection occurs. Furthermore, what mix of susceptibilities ultimately emerges is probably rather sensitive to initial conditions of antimicrobial agent, species, strain and exposure.

For heavy metals, associations have been identified between reduced zinc or copper susceptibility, serovar and antibiotic resistance among pig *Salmonella* isolates, including monophasic *Salmonella* Typhimurium. These are established foodborne pathogens. Furthermore, there is reasonable evidence of a co-resistance phenomenon involving copper, macrolides and perhaps vancomycin among enterococci of pigs. However, the relevance of this to disease-causing strains in humans remains undetermined.

LA-MRSA shows some evidence of co-resistance consequent on selection by zinc, but this selective pressure appears to be modest in the face of age-related reductions in prevalence of the organism among pigs. How the effect of zinc compares with selection by antibiotics of this multi-resistant organism is unclear, given that LA-MRSA ST398 isolates are frequently resistant to the commonly-used tetracyclines. There is some evidence that QAC biocides might also select for MRSA carriage in nursery-age pigs. Whether selection, by zinc or QAC, at this age may affect the risk of LA-MRSA contamination of foodstuffs is currently unclear. By contrast in *L. monocytogenes*, a species notable for little antibiotic resistance, reduced biocide susceptibility in environmental field strains doesn’t appear typically to be associated with changes in antibiotic sensitivity.

## 6. Field Observations *vs.* Laboratory Findings

There is a considerable amount of experimental evidence, as discussed in Section 4 and Section 5, for co-selection of antibiotic resistance by exposure of common primary and opportunistic bacterial pathogens, to biocides principally, and also to heavy metals. However, this is not matched by compelling evidence of similar effects in farm and other environments regularly exposed to such agents. Nonetheless, biocide susceptibility is commonly measured in terms of MIC and several experimental studies have shown that exposure to biocides, particularly compound products, may result in elevated antibiotic MIC (including the MAR phenotype among *Salmonella*) without significant change in MIC of the biocide. Thus, there may be some effects of biocides on antibiotic sensitivity among field strains where the association is not evident from testing of MIC values. Further testing, of kill times and of effects of biocides against organisms in stressed states, on surfaces, in biofilms, *etc.*, might reveal more associations than are currently evident. Evidence [150] that the *sil* operon improves copper handling by monophasic *S.* Typhimurium only under anaerobic conditions, such as are encountered in the gut, also indicates that test conditions may substantially affect which associations are seen between heavy metals and antibiotics.

Nonetheless, there are some reasons to be sceptical about the occurrence in real-world environments of phenomena described in laboratory studies. Controlled, extended laboratory exposure of bacterial strains to sub-lethal concentrations of antimicrobial substances is not likely to be a good model for interactions between biocides and organisms in the environment. In-use conditions usually involve transient high-level application, often of a synergistic compound preparation, followed by variable exposure of survivors to residual concentrations. Some biocides are persistent, whilst others biodegrade rapidly, and the effectiveness of others is highly-dependent on dilution, pH or interfering organic substances [38].

This variation in effective exposures, given the differences seen even between similar laboratory studies of controlled exposure, is likely to yield unpredictable patterns of altered susceptibility that may also include increased sensitivity to some agents. Certainly, some biocides and heavy metals can have a counter-selective effect on antibiotic resistance genes and/or phenotypes [6,94,177,178]. Furthermore, certain effects observed in the laboratory may not be as adaptive as they initially appear. For example, the induction of efflux by triclosan binding to the repressor of the SmeDEF multi-drug transporter of *S. maltophilia* only occurs once triclosan is present at toxic levels [64,179].

Even once significantly reduced susceptibilities to biocide, metal or antibiotic have been generated, the fitness costs discussed previously will militate against survival of many adapted strains in the context of shifting and re-establishing bacterial communities such as is likely in farm environments. Nonetheless some adaptations, such as biofilming capacity, may prove immediately advantageous for survival and there are agents (particularly heavy metals) and circumstances, such as prevalent antibiotic use, where a selective pressure may be maintained for a strain following adaptation for reduced susceptibility. However, the particular circumstances where an appropriate selective pressure is applied at the right time to maintain or intensify a particular reduction in antibiotic sensitivity that has been generated by biocide use may not be especially common. Indeed, instead of co-selection, biocides and antibiotics can have additive or synergistic antimicrobial effects when applied together [180,181].

In cases of heavy metal contamination, the antimicrobial selective pressures are likely to be sub-lethal and exerted for a long time, extending to many years in the case of contaminated soil or sediment. It is of interest that investigations into heavily copper-supplemented pigs and heavily contaminated water or soil have provided some of the most convincing data on associations between reduced susceptibility to antibiotic and non-antibiotic (*i.e.*, metal) antimicrobials, in a wide range of bacteria.

## 7. Conclusions

The long history of biocide use in healthcare, agriculture, industry and elsewhere has not yielded any consistent evidence of a generalised problem of reduced susceptibility to such agents. Furthermore, the comparatively brief history of antibiotic use and the rapid emergence of single and multiple antibiotic resistance among exposed bacteria has provided compelling evidence of a causal association between antibiotic consumption and the occurrence of antibiotic resistance. This drives efforts to control medical and veterinary use of antibiotics. Increasing hygiene measures and, in stock rearing, substitution of prophylactic and growth-promoting antibiotics with heavy metal salts has been part of the response to greater constraints on antibiotic use.

Concerns have been raised in recent years that known and suspected links between biocide or heavy metal use and antibiotic resistances may prove significant in perpetuating those resistances. The legitimacy of these concerns has been bolstered by evidence from antibiotic and biocide research that co-selection may yield multiple resistances and that even low-level reduced antibiotic sensitivity can foster the development of high-level resistance given a suitable selective pressure. The heavy metals are one group of agents that, by deliberate application or by contamination, are typically present at non-lethal concentrations and for prolonged periods compared with biocides. In this respect their interactions with bacteria more closely resemble those of antibiotics than of disinfectants or antiseptics.

There is much evidence from laboratory studies that exposure of bacteria to members of one class of antimicrobial agent can affect susceptibilities to other classes. However, data from hospital and other healthcare environments has provided conflicting evidence as to the likely clinical significance of co-selection of antibiotic resistant pathogens by biocides. Data for similar effects on the diverse microbiota on farms and in the food chain is yet more limited than for healthcare-associated pathogens. Again, laboratory studies indicate that such links exist for the important zoonotic pathogens but survey data suggests that, for now, counter-selective processes largely limit the effects of co-selective pressures. For heavy metals, in addition to identified genetic linkages with antibiotic resistance determinants, evidence exists from animal and environmental studies for links between the use of such agents in the pig and poultry industries and increased genetic mobility and co-selection. However, the immediate significance of susceptibility associations in respect of foodborne pathogens is uncertain.
